# Selective inhibition of jasmonic acid accumulation by a small α, β-unsaturated carbonyl and phenidone reveals different modes of octadecanoid signalling activation in response to insect elicitors and green leaf volatiles in *Zea mays*

**DOI:** 10.1186/1756-0500-4-377

**Published:** 2011-10-03

**Authors:** Jurgen Engelberth

**Affiliations:** 1 Department of Biology, University of Texas at San Antonio, One UTSA Circle, San Antonio, TX 78249, USA

## Abstract

**Background:**

Plants often release a complex blend of volatile organic compounds (VOC) in response to insect herbivore damage. Among those blends of VOC green leaf volatiles (GLV) have been demonstrated to function as defence signals between plants, thereby providing protection against impending herbivory. A problem in understanding the mode of action of these 6-carbon aldehydes, alcohols, and esters is caused by their structural diversity. Besides different degrees of oxidation, *E*-2- as well as *Z*-3-configured isomers are often released. This study was therefore initiated to determine the structural requirement necessary to exhibit biological activity measured as jasmonic acid (JA) accumulation in *Zea mays *seedlings.

**Findings:**

The structure/function analysis of green leaf volatiles and related compounds revealed that an olefinic bond in position 2 or 3 and a size of 6-8 carbons is required for biological activity in maize. Also, it was found that the presence of an α, β-unsaturated carbonyl is not a prerequisite for activity. However, by treating plants first with volatile acrolein it was discovered that this smallest α, β-unsaturated carbonyl inhibits JA accumulation in response to insect elicitor treatment, but not after GLV exposure. This selective inhibitory effect was also found for phenidone, an inhibitor of lipoxygenases. These findings led to the discovery of a pool of protein-associated 12-oxo-phytodienoic acid, a biosynthetic precursor of JA, which appeared to be rapidly converted into JA upon exposure to GLV.

**Conclusions:**

The structure/function analysis of GLV demonstrates a high degree of correlation between the compounds released by wounded plants in nature and their biological activity. The selective inhibitory effects of acrolein and phenidone on insect elicitor- and GLV-induced JA accumulation in maize led to the discovery of a pool of protein-associated precursor, which is rapidly activated and transformed to JA after exposure to GLV. This novel mechanism for JA accumulation sheds new light on the biosynthetic variability of the octadecanoid signalling pathway and explains the observed differences in the response of maize seedling to inhibitors of JA accumulation.

## Background

The ecological and physiological functions of herbivore-induced volatiles have become widely accepted not only as signals in tritrophic interaction, but also as signals that communicate impending herbivory between plants. In this context, certain VOC generally referred to as green leaf volatiles (GLV) were shown to prepare or prime receiver plants against these pests rather than inducing effective direct defences [1,2]. GLV, which mainly consist of 6-carbon aldehydes, alcohols, and their esters, are derived from the hydroperoxides of polyunsaturated fatty acids through the hydroperoxide lyase (HPL) pathway [3]. Besides *Z*-3-configurated compounds the respective *E*-2 isomers are also often released immediately after mechanical damage or insect herbivory. Interestingly, Allmann and Baldwin [4] found that when *Manduca sexta *larvae were feeding on *Nicotiana attenuata*, heat-labile factors in the caterpillar's saliva isomerised *Z*-3-configured GLV into their respective *E*-2-isomers, which in turn attracted natural enemies of the caterpillar. However, maize (*Zea mays*), like many other plants, mostly released the *Z*-3-configurated GLV immediately after being mechanically damaged [1]. The responses of plants to all these different isomers, as it is described above, raised the question of structural requirements that are necessary to exhibit biological activity. It has been suggested that the presence of an α, β-unsaturated carbonyl, as it occurs in *E*-2-configured aldehydes, might be such an active motif. But, as already pointed out in a study by Heil *et al *[5], many of the active compounds do not have this structural feature. Also, considering the swiftness of GLV activity in maize [6], it is rather unlikely that 6-carbon alcohols and their respective esters are first hydrolysed (for the esters) and then dehydrogenated before biological functionality is achieved. For maize, natural occurring GLV were shown to significantly induce jasmonic acid (JA) accumulation [1,6]. In those studies *Z*-3- as well as *E*-2-configurated GLV were found to be equally active.

This study was therefore initiated to provide a better understanding towards the structural diversity required for biological activity of naturally occurring GLV and related compounds. A wide array of compounds including selected α, β-unsaturated carbonyls of varying sizes were tested with JA accumulation serving as a marker for activity. Besides providing data on the structural requirements needed to activate JA accumulation in maize as our model plant this study also presents evidence for the selective inhibition of JA accumulation by small α, β-unsaturated carbonyls. Additionally, the existence of a novel type of stored precursor for JA was revealed, presumably 12-oxo-phytodienoic acid (OPDA), which appears to be rapidly transformed into JA upon exposure to GLV.

## Results and discussion

The structural requirements of green leaf volatiles (GLV) and related compounds to induced JA accumulation in maize seedlings were investigated in the present study. Besides the common *Z*-3- and *E*-2-configurated 6-carbon aldehydes, alcohols, and esters an array of selected α, β-unsaturated carbonyls of varying sizes were also analyzed. The naturally occurring *Z*-3- and *E*-2- GLVs were found to be equally active with regard to JA induction. For example, Z-3-hexenal induced 45.3 ± 12.2 (standard deviation) ng/gFW JA and *E*-2-hexenal induced 64.7 ± 16.7 ng/gFW JA (Additional file 1: Table S1). Likewise, accumulation of JA induced by *Z*-3-hexenol, *E*-2- hexenyl acetate, and Z-3-hexenyl acetate did not differ significantly (Table S1). Additionally, activity was also found for *E*-2-octenal (26 ± 9.7 ng/gFW), which was however significantly lower than that of the 6-carbon compounds. Interestingly, *E*-5-hexenyl acetate did not exhibit any activity towards JA accumulation, which was in contrast to a study published by Heil *et al *[5], who described *E*-5-hexenyl acetate as equally active as other GLV in extra floral nectar induction in lima beans. With regard to size it was found that 6-8 carbon compounds were active, whereas smaller (≤ 4-carbon) and also larger molecules (e.g. *E*-2-nonenal) showed no activity. Additionally, a saturated 6-carbon compound (hexanol) was also inactive. This correlated well with previously published results by Farag *et al *[7], who also found that 6-carbon compounds had the highest activity, while neither 5-carbon nor 7-carbon compounds exhibited any activity. These results suggest that a.) the olefinic bond has to be either in position 2 or 3, and b.) the required size necessary to activate JA accumulation lies between 6- and 8-carbons.

It has been suggested that an α, β-unsaturated carbonyl might be the structural feature responsible for the activity of GLV. However, the results in Table S1 clearly show that this is an unlikely hypothesis. Acrolein, *E*-2-butenal, *E*-2-nonenal, and *cis *jasmone were inactive with regard to JA accumulation. Likewise, the *E*-2-configured 6-carbon aldehyde was as active as its *Z*-3 counterpart. Based on these data it can be concluded that an α, β-unsaturated carbonyl is not a structural requirement for activity.

**Figure 1:**
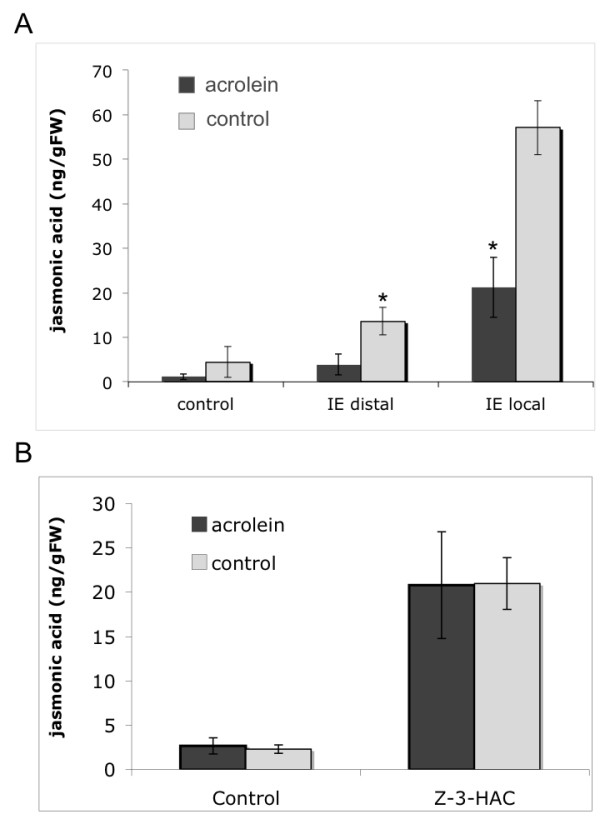
**Differential effect of acrolein on insect elicitor- and *Z*-3-hexenyl acetate- induced jasmonic acid accumulation**. A. Effect of acrolein pre-treatment on insect elicitor (IE)-induced jasmonic acid accumulation in maize seedlings. Jasmonic acid was measured in the local (directly treated) and the distal (leaf upwards from the treatment site) section of the same leaf. B. Effect of acrolein pre-treatment on *Z*-3-hexenyl acetate (*Z*-3-HAC)- induced jasmonic acid. Jasmonic acid was measured 60 min after insect elicitor treatment and 30 min after *Z*-3-HAC exposure. Error bars represent standard deviation. An * indicates a significant difference between acrolein-treated and control plants (*t*-test, p ≤ 0.05).

As shown previously, certain small α, β-unsaturated carbonyls like malonyl dialdehyde and acrolein were powerful inducers of defensive genes, in particular in the absence of jasmonic acid [8]. Although acrolein was found to have no effect on JA accumulation it could still act synergistically with other treatments. Therefore, the effect of acrolein on insect elicitor- and *Z*-3-hexenyl acetate-induced JA accumulation was tested. It was found that acrolein significantly inhibited JA accumulation after treatment with insect elicitors (IE) (Figure 1A). Not only was the local response reduced by 60%, but also the distal accumulation of JA (70% reduction), which was previously shown to be characteristic for IE activity [6]. In contrast to these results no inhibitory effect of acrolein on GLV-induced JA accumulation was found (Figure 1B). This selective inhibition of IE-induced JA accumulation clearly suggested that at least different modes for JA accumulation exist in maize, which are differentially activated by these stimuli. This was unexpected since both treatments, GLV exposure and IE application, exhibited similarities with regard to JA accumulation. For example, in both cases (for IE application in the distal part of the treated leaf) JA accumulation was not associated by a concomitant increase of 12-oxo-phytodienoic acid (OPDA) [6].

**Figure 2:**
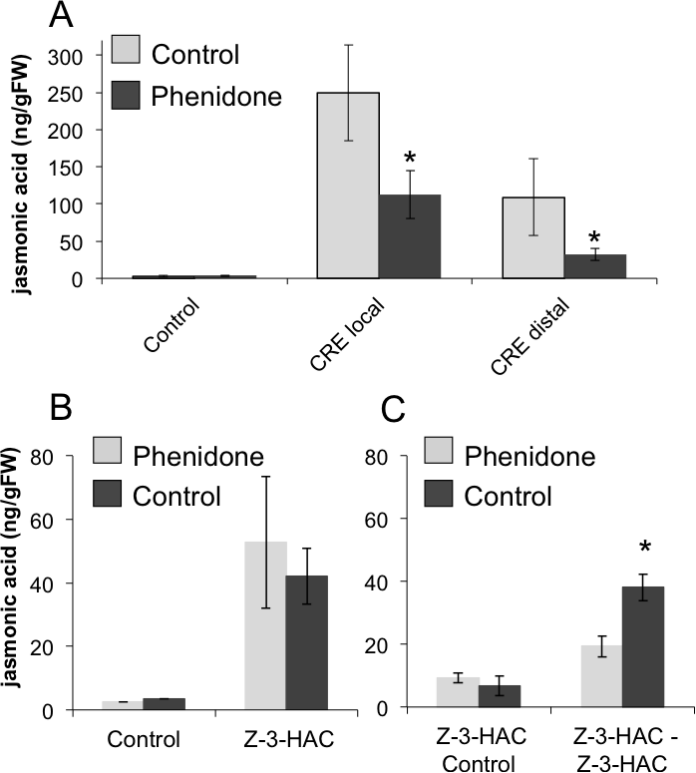
**Effects of phenidone on insect elicitor- and *Z*-3-hexenyl acetate-induced jasmonic acid accumulation**. Maize seedlings were treated overnight with phenidone and then induced by treatment with insect elicitor (IE) or exposure to *Z*-3-hexenyl acetate (*Z*-3-HAC). Jasmonic acid was measured 60 min after IE treatment and 30 min after *Z*-3-HAC exposure. A. Effect of phenidone on IE- induced jasmonic acid. Jasmonic acid was measured in the local (directly treated) and the distal section of the same leaf. B, C. Effect of phenidone on *Z*-3-HAC-induced jasmonic acid. B. Overnight treatment with phenidone had no effect on *Z*-3-HAC-induced JA accumulation when compared to similarly treated control plans. C. Maize seedling were first exposed to *Z*-3-HAC for 30 min and then treated with phenidone overnight. Controls were not exposed to *Z*-3-HAC, but also treated with phenidone overnight. After 15 h treatment with phenidone both treatment groups were exposed to *Z*-3-HAC. A significant reduction of jasmonic acid accumulation was found only in maize seedlings that were exposed to *Z*-3-HAC before phenidone treatment. Error bars represent standard deviation. An * indicates a significant difference between phenidone-treated and their respective control plant (*t*-test, p ≤ 0.05).

**Figure 3:**
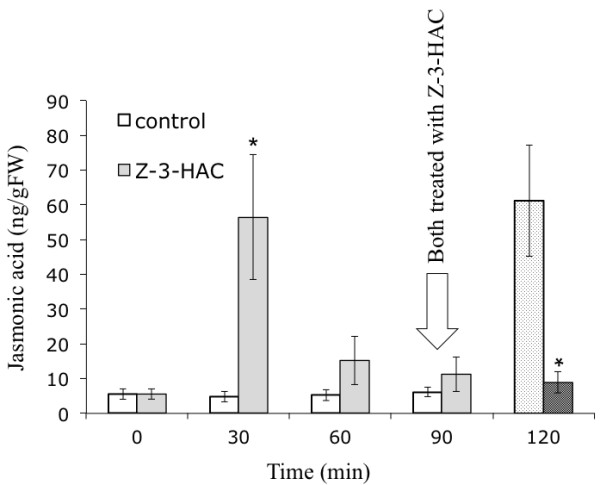
**Repeated exposure to *Z*-3-hexenyl acetate does not induce more jasmonic acid**. Maize seedlings were exposed to *Z*-3-hexenyl acetate (*Z*-3-HAC) at time point 0 and jasmonic acid accumulation monitored over 90 min (grey bars). After reaching baseline levels seedlings were again exposed to *Z*-3-HAC and JA measured 30 min later (dotted grey bar). No further accumulation was measured. At the same time control seedlings (white bars) did respond normally to *Z*-3-HAC exposure (white bar with dots). Error bars represent standard deviation. An * indicates a significant difference between *Z*-3-HAC-treated and control plants (*t*-test, p ≤ 0.05).

**Figure 4:**
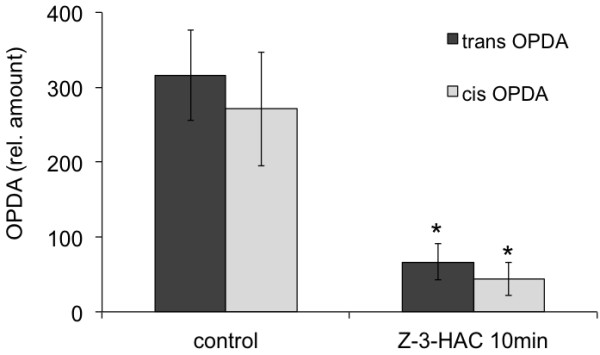
**Detection of 12-oxo-phytodienoic acid (OPDA) in a protein fraction of corn leaves**. OPDA was detected in a crude protein fraction isolated from maize leaves. 10 min after exposure to *Z*-3-hexenyl acetate (*Z*-3-HAC) almost 80% of the protein-associated OPDA disappeared and was presumable transformed into jasmonic acid. Error bars represent standard deviation. An * indicates a significant difference between *Z*-3-HAC-treated and control plants (*t*-test, p ≤ 0.05).

To further characterize the differential induction and accumulation of JA in maize we chose a pharmacological approach by using phenidone, a highly specific inhibitor of lipoxygenases (LOX) [9-11], which catalyze the first step in the biosynthesis of JA. After overnight incubation of maize seedlings with phenidone, IE-induced JA in the local and distal part of the treated leaf was reduced 57% and 82%, respectively (Figure 2A). In contrast, GLV-induced JA production was not affected by phenidone, as it has been described for acrolein above (Figure 2B). However, when maize seedlings were first exposed to *Z*-3-hexenyl acetate for 1 h and then treated with phenidone for 15 h, they showed a significant reduction of JA accumulation in response to a second exposure to *Z*-3-hexenyl acetate (17.4 ng/g FW) when compared to maize seedlings that were not first exposed to *Z*-3-hexenyl acetate before phenidone treatment (38.1 ng/g FW) (Figure 2C). This also implied that once the pool of the presumed precursor is emptied GLV should not be able to induce another transient induction of JA. This was confirmed by repeated exposure to GLV as shown in Figure 3. While a first treatment of maize seedlings with *Z*-3-hexenyl acetate induced normal levels of JA (56.5 ng/gFW), a second exposure to *Z*-3-hexenyl acetate 90 min after the first did not induce any JA accumulation anymore. Taken together, the selective inhibition of JA accumulation by acrolein and phenidone strongly suggested that GLV-induced JA was not entirely synthesized *de novo*, but rather utilized a pool of precursors, which had to be positioned downstream of 13-hydroperoxy linolenic acid. This pool appeared to be rapidly activated by exposure to GLV and transformed into JA. Once emptied, the pool was filled-up again through *de novo *synthesis as indicated by the inhibitory effects of phenidone (Figure 2C). But what was the nature of this pool of stored octadecanoids, which was rapidly activated upon exposure to GLV? To answer this question maize lipids were first tested for the abundance of lipid-bound precursors of JA, either by acid hydrolysis or the use of lipases. However, no potential precursor could be identified (data not shown). But when a crude protein fraction from maize leaves was heated and subsequently extracted for the abundance of oxylipins, we found a significant portion of both, *cis*- and *trans*-OPDA associated with the protein (Figure 4). More importantly, when maize plants were first exposed to Z-3-hexenyl acetate for 10 min and then tested for protein-associated OPDA, this pool was significantly reduced (Figure 4). This experiment provided first evidence on the nature of the precursor, which may be rapidly converted into JA upon exposure of plants to these volatile signals. It has to be mentioned that this pool appears to be very labile. Leaving the OPDA-containing protein fraction at room temperature for 2-3 min already removed all of the bound OPDA from the protein, presumable due to the presence of GLV during the extraction. This makes it extremely difficult to isolate, identify, and characterize the putative OPDA binding protein. Therefore, a technique has to be developed that stabilizes this complex and allows for the concomitant isolation of the protein with its ligand still attached to it.

## Conclusion

The structure/function analysis of GLV and related compounds revealed a certain flexibility in the structural requirement for activity. A size of 6 carbons and an olefinic bond in position 2 or 3 appear to be the only common features among the active compounds tested. This may seem rather unspecific. However, when we look at GLV as plants release them in response to insect herbivory, it becomes obvious that the response to these volatiles correlates well with this profile. This may be the reason for the relatively wide activity range of those structures and would allow for the plant to recognize the whole diversity of potential GLV-related compounds as they are emitted from most plant species upon insect herbivore damage.

**Figure 5:**
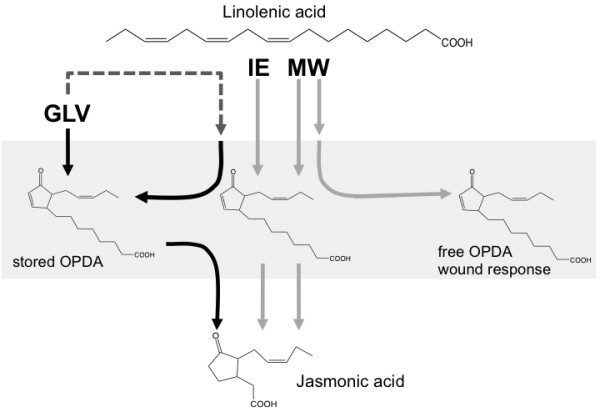
**A model describing the different modes of jasmonic acid (JA) accumulation in maize seedlings in response to insect elicitor treatment, mechanical wounding, and exposure to green leaf volatiles**. Insect elicitors (IE) and green leaf volatiles (GLV) induce JA accumulation without the concomitant induction of free 12-oxo- phytodienoic acid (OPDA) [6]. In contrast, mechanical wounding (MW) also induces the accumulation of free OPDA [6]. But while IE- and MW-induced JA is synthesized *de novo *(grey arrows) through the complete octadecanoid pathway, GLV-induced JA appears to be derived from a pool of protein- associated OPDA in the leaves (black arrows). Restoring this pool of protein-bound OPDA requires the activation of the first part of the octadecanoid pathway through a yet unknown mechanism (dashed line).

Although GLV and IE induce JA accumulation without the concomitant accumulation of OPDA, significant differences were observed with regard to the inhibitory effects of acrolein and phenidone. For both compounds an inhibitory effect was found for IE-induced JA accumulation, whereas GLV activity was not affected. This suggested a pool of precursors, which is rapidly activated upon GLV recognition. Such a pool was identified as OPDA within the protein fraction. A model that summarizes these finding is provided in Figure 5. While IE- and mechanical wounding (MW) induced the accumulation of JA through activation of the whole biosynthetic pathway beginning with linolenic acid, GLV appeared to activate a stored precursor, which was presumable associated with a protein fraction.

Identification of the protein may provide further insights into the mechanistics of GLV-induced JA accumulation and may further provide a suitable target for agricultural applications of GLV-induced priming.

## Material and methods

### Chemicals

*Z*-3-hexenal (50% in triacetin), *E*-5-hexenyl acetat, Acrolein, *E*-2-butenal, *E*-2-octenal, hexanol, and *E*-2-nonenal were purchased from Sigma-Aldrich. *Z*-3-hexenol, *Z*-3-hexenyl acetate, *E*-2-hexenal, *E*-2-hexenol, *E*-2-hexenyl acetate, dihydro jasmonic acid-methyl ester, and *cis *jasmone were obtained from Bedoukian (Bedoukian Research, Danbury, CT). Dihydro jasmonic acid-methyl ester was converted to dihydro jasmonic acid (dhJA) by alkaline hydrolysis and used as the internal standard for JA quantification [1]. All solvents used were analytical grade.

### Plant material

Maize seeds (*Zea mays *var. Kandy King) were purchased from J.W. Jung Seed Co. Randolph, WI, and grown as described previously [12]. 10-12 day old plants were used for the experiments described below. At that time plants were at the V2 stage.

### Plant treatments

To measure the short-term production of JA, intact maize plants (receiver plants) were exposed to the chemicals listed in Table S1 in 7 l glass cylinders. 100 μg of each compound (dissolved in dichloromethane, 10 μg/μl) were pipetted onto a cotton ball in the glass cylinder. Controls consisted of a plant in a chamber with 100 μl of pure dichloromethane applied onto a cotton ball. Plants were exposed to these chemicals for 30 min and the second leaf of each seedling was harvested and immediately frozen in liquid N_2 _for further processing.

Preparation of insect elicitor (IE) from larvae of BAW was done as described previously [6]. For induction with IE an area of about 2 mm × 10 mm on the third leaf of intact maize plants was scratched with a razor blade and 10 μl of IE from BAW were immediately added to the wounded site. For wounding, an area of about 2.5 cm long was scratched at four positions, two on each side of the midrib. For controls, buffer only was added to the wounded site. Sections of about 2.5 cm were taken from the wounded site (local) and distal from there and immediately shock-frozen in liquid N_2_. These leaf sections were then analyzed for JA accumulation.

### Pharmacological effects of phenidone and acrolein on JA accumulation

To study the pharmacological effects of phenidone on JA accumulation in maize seedlings that were treated either with IE or GLV, plants were cut at their base and immediately transferred into vials with either 2 mM phenidone in water or water as a control. After overnight incubation plants were treated the next day either by exposing them to Z-3-6:AC (for 30 min) or by treatment with IE (for 60 min). As described above, leaf segments were analyzed for JA accumulation.

In a variation of these experiments we exposed one set of plants to *Z*-3-hexenyl acetate first for 1 h in a 7 l glass cylinder as described above. The cylinders were then removed and the plants were allowed to rest for one additional hour. Plants were then cut and treated with phenidone or water as described above. Control plants were treated the same way, except that initially no *Z*-3-hexenyl acetate was applied to the plants.

For treatment with acrolein maize seedlings were first exposed to 1 mg of pure acrolein in a 7 l glass cylinder for 1 h. Plants were then removed from the cylinder and allowed to rest for 30 min at ambient air in the hood to remove excess amounts of acrolein. Plants were then treated with IE or *Z*-3-hexenyl acetate as described above. Leaf segments from the second leaf were then taken and analyzed for JA accumulation as described below.

### Quantification of jasmonic acid and phytodienoic acid

Extraction and quantification was performed as described previously [1,13]. In brief, plant tissues were frozen in liquid N_2 _and about 100 mg of each sample was transferred to 2 ml screw cap FastPrep^® ^tubes (Qbiogene, Carlsbad, CA) containing 1 g Zirmil™ beads (1.1 mm; SEPR Ceramic Beads and Powders, Mountainside, NJ). DhJA (100 ng) was added to the 2 ml tubes prior to sample addition as the internal standard. The samples were mixed with 300 μl of 1-propanol:H_2_0:HCl (2:1:0.002) and shaken for 30 s in a FastPrep^® ^FP 120 tissue homogenizer (Qbiogene, Carlsbad, CA). Dichloromethane (1 ml) was added to each sample, re-shaken for 10 s in the homogenizer, and centrifuged at 11,300 × *g *for 30 s. The bottom dichloromethane:1-propanol layer containing jasmonic acid and other plant hormones was then transferred to a 4 ml glass screw cap vial. The organic phase was evaporated by a constant air-stream and 100 μl of diethyl ether: methanol (9:1, vol: vol) added. Carboxylic acids were converted into methyl-esters by the addition of 2 μl of a 2.0 M solution of trimethylsilyldiazomethane in hexane. The vials were then capped, vortexed, and allowed to sit at room temperature for 30 min. Excess trimethylsilyldiazomethane was then destroyed by adding an equivalent molar amount of acetic acid to each sample.

Volatile metabolites were separated from the complex mixture by vapor phase extraction as described in [1,13]. The trapped volatiles were then eluted with 150 μl dichloromethane and analyzed by CI-GC/MS [1,13]. Quantification was based on the internal standard and the fresh weight of the plant material.

### Characterization of an internal protein-associated pool of 12-oxo-phytodienoic acid (OPDA)

Maize seedlings were exposed to 1 mg *Z*-3-hexenyl acetate in a 7 l glass container. Controls were also placed in a glass container, but without the addition of Z-3-hexenyl acetate. After 10 min exposure plants were removed from the container and a 2.5 cm segment of the second leaf was taken for analysis and shock-frozen in liquid N_2 _in 2 ml screw cap vials. To extract protein associated octadecanoids, 1 g of Zirmil beads, 1 ml DCM and 500 μl potassium phosphate buffer (KPi) (50 mM, pH 6.5) were added to the frozen plant material in the vial and the tissue was disrupted as described above for regular hormone extraction. After centrifugation at 10,000 × g for 1 min, 500 μl of the upper water phase were taken and mixed with 1.5 ml of ice cold methanol. The sample was vortexed and incubated at -20°C for 1 h. After centrifugation for 3 min at 10,000 × g the supernatant was discarded and the pellet re-dissolved in 200 μl of KPi buffer and 200 μl of the regular hormone extraction solution (see above). Also, 50 ng of internal standard (dhJA) were added. The sample was then incubated at 95°C for 10 min, cooled down on ice, and then extracted for hormones as described above by adding 1 ml DCM. Because appropriate internal standards were not commercially available, OPDA was quantified by comparison with dhJA and is therefore plotted as relative amounts.

### Statistical analysis

At least three biological replicates of all experiments were performed. Data were analyzed for significance with *t *test (p < 0.05). Treatments were compared to appropriate controls.

## Competing interests

The authors declare that they have no competing interests.

## Authors' contributions

JE designed and performed the experiments, performed the statistical analysis, and wrote the manuscript.

## Supplementary Material

Additional file 1 **Table S1**. Activity and structures of green leaf volatiles and related compounds. Click here for file
