# Supplementary material for: Selective inhibition of jasmonic acid accumulation by a small α, β-unsaturated carbonyl and phenidone reveals different modes of octadecanoid signalling activation in response to insect elicitors and green leaf volatiles in Zea mays
Source: BMC Res Notes. 2011 Oct 3;4:377. doi: 10.1186/1756-0500-4-377 (PMC3192698; doi:10.1186/1756-0500-4-377)
Supplement: Additional file 1 — Table S1. Activity and structures of green leaf volatiles and related compounds. [file 1756-0500-4-377-S1.DOC]

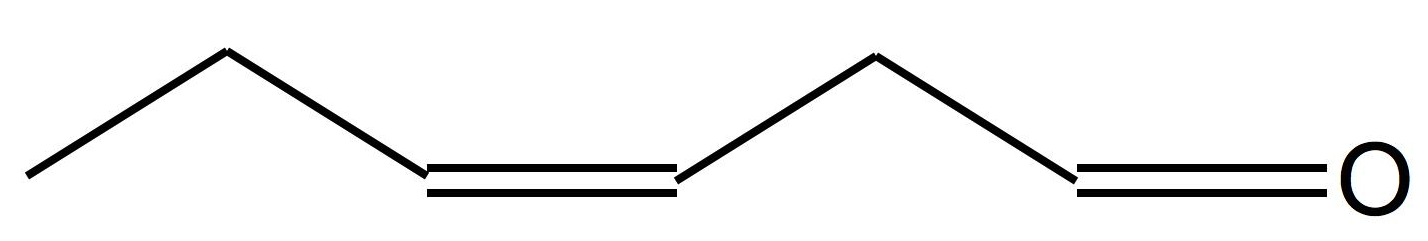

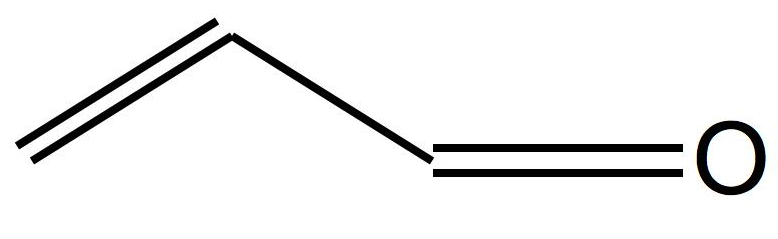

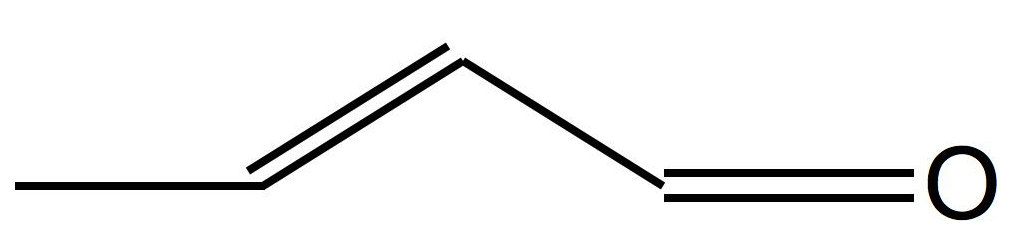

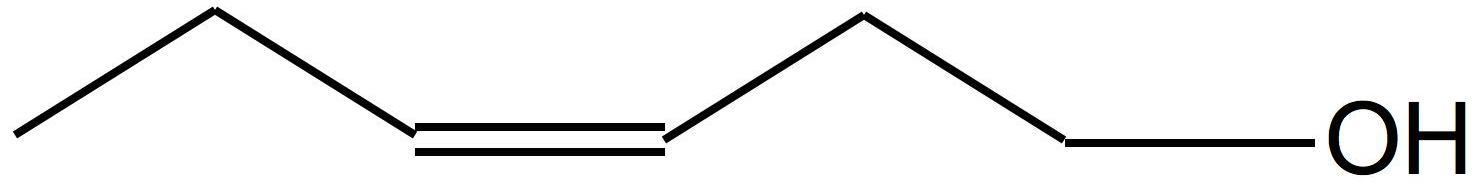

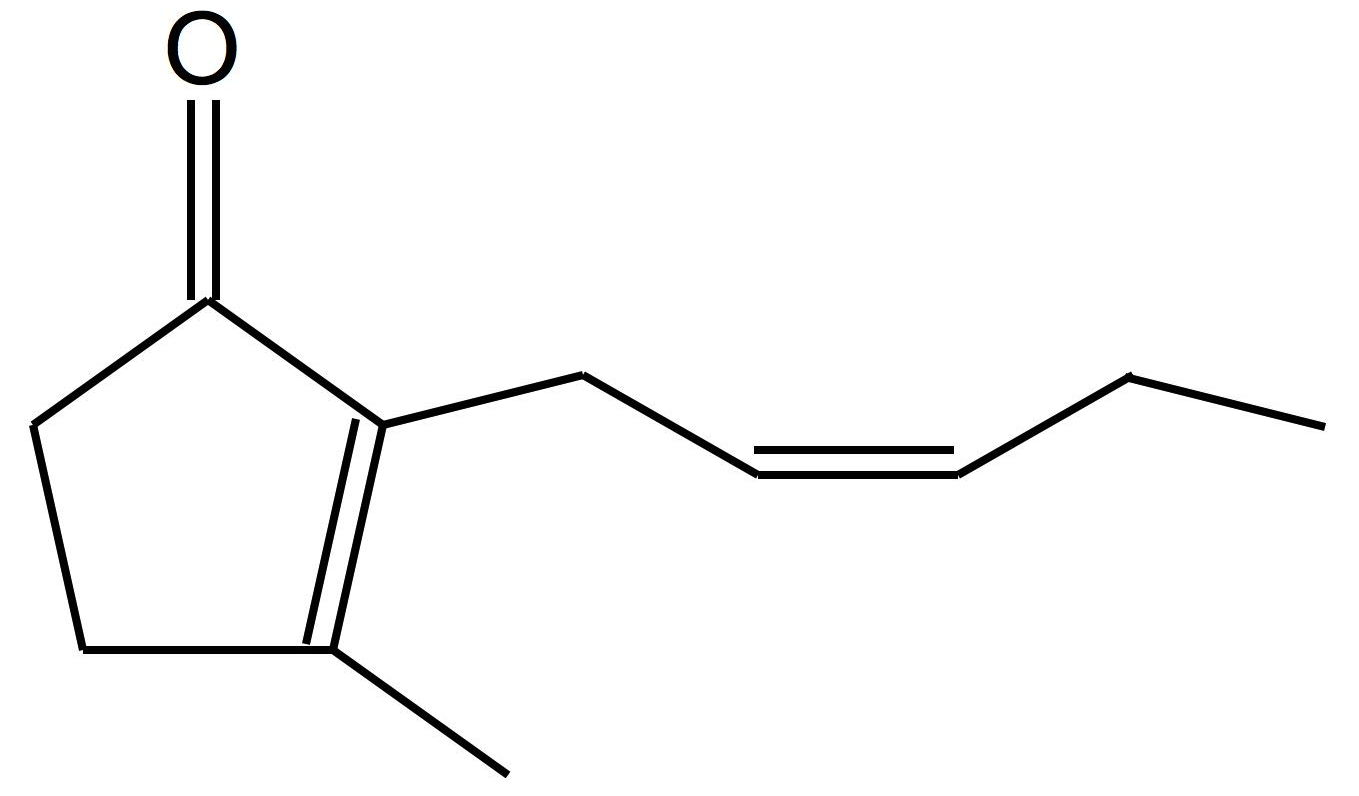

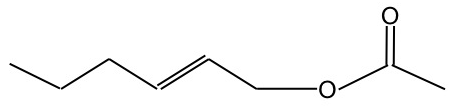

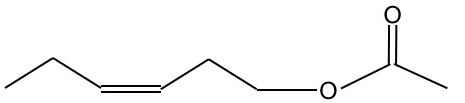

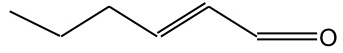

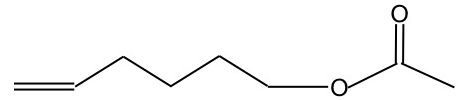

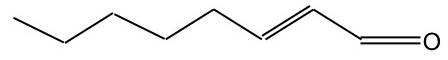

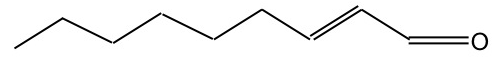

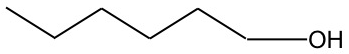


All compounds were applied pure at 1 mg/7 l volume. Activity is expressed as total jasmonic acid (JA) ± standard deviation (SD) after 30 min of exposure. All experiments were performed with 4 biological replicates. An * indicates a significant difference between treated and control plants (*t*-test, p≤0.05).
